# Enhanced Sensitivity of a Modified Quaking‐Induced Conversion Diagnostic Test for the Broad Detection of Sporadic and Inherited Prion Diseases: A Retrospective Study

**DOI:** 10.1002/ana.78162

**Published:** 2026-01-26

**Authors:** Jennifer Myskiw, Rebecca Fox, Dominic M.S. Kielich, Lise Lamoureux, Melanie Leonhardt, Olivia Nykvist, Jessy A. Slota, Kristen Avery, Clark Phillipson, Kathy Frost, Sharon Simon, Brian S. Appleby, Ben A. Bailey‐Elkin, Stephanie A. Booth

**Affiliations:** ^1^ Mycobacteriology, Vector‐borne and Prion Diseases Division, National Microbiology Laboratory, Public Health Agency of Canada Winnipeg MB Canada; ^2^ Department of Medical Microbiology and Infectious Diseases, Faculty of Health Sciences University of Manitoba Winnipeg MB Canada; ^3^ National Prion Disease Pathology Surveillance Center, Case Western Reserve University School of Medicine Cleveland OH; ^4^ Department of Neurology, Psychiatry, and Pathology Case Western Reserve University School of Medicine/University Hospitals Cleveland Medical Center Cleveland OH

## Abstract

**Objective:**

Quaking‐induced conversion (QuIC) tests, which detect prion‐seeding activity in cerebrospinal fluid (CSF), have markedly advanced the antemortem diagnosis of prion diseases such as Creutzfeldt‐Jakob disease (CJD). These tests provide high diagnostic accuracy and enable timely differentiation from other rapidly progressive neurodegenerative disorders. However, a key limitation of current QuIC tests are the reduced sensitivity in detecting inherited prion diseases and rare sporadic subtypes, including variably protease‐sensitive prionopathy (VPSPr). To address this gap, we evaluated a simplified QuIC test, end‐point QuIC (EP‐QuIC), incorporating a novel recombinant prion protein substrate derived from the North American deer mouse (*Peromyscus maniculatus*).

**Methods:**

The diagnostic performance of the modified QuIC test was evaluated using CSF samples from 61 sporadic CJD, 50 inherited prion disease, and 5 VPSPr cases.

**Results:**

EP‐QuIC with the deer mouse substrate achieved 96.6% sensitivity (111/116) and 100% specificity (35/35), outperforming both standard EP‐QuIC (87.1%) and next‐generation (IQ‐CSF) real‐time‐QuIC (72.4%) across the same cohort. Notably, this enhanced assay detected inherited mutations, such as D178N, that were previously undetectable with existing diagnostic tests.

**Interpretation:**

These findings demonstrate that adapting EP‐QuIC with an optimized substrate, termed enhanced sensitivity QuIC (ES‐QuIC), significantly improves diagnostic performance for inherited and atypical prion diseases. By expanding the diagnostic reach of QuIC tests, this study strengthens antemortem surveillance, reduces reliance on postmortem confirmation, and improves opportunities for early intervention and clinical trial enrollment, particularly for genetic cases most likely to benefit from emerging therapeutic strategies. ANN NEUROL 2026;99:1303–1314

Human prion diseases are rare, fatal neurodegenerative disorders caused by the accumulation of misfolded prion proteins (PrP^Sc^). Although all share similar brain pathology, clinical phenotypes can vary and resemble other neurological conditions, complicating antemortem diagnosis. This variability reflects different disease etiologies: sporadic (85–90% of reported cases), genetic (10–15%), and acquired (<1%) as well as different “strains” within each etiologic group.^1^ Sporadic Creutzfeldt‐Jakob disease (sCJD) alone encompasses 6 distinct molecular strains (MM1/MV1, VV1, VV2, MV2, MM2C, and MM2T), each with characteristic clinicopathological features.^2^ Other sporadic forms, such as variably protease‐sensitive prionopathy (VPSPr), display unique biochemical and clinical traits.^3^ Inherited prion diseases (IPDs) also exhibit broad phenotypic diversity, ranging from sCJD‐like presentation to protracted neurodegenerative courses extending over several years.^4^, ^5^

The quaking‐induced conversion (QuIC) test has revolutionized the differential diagnosis of human prion diseases. This test detects the presence of PrP^Sc^ by using patient cerebrospinal fluid (CSF) to seed the formation of recombinant prion protein (rPrP) amyloids, monitored through Thioflavin T (ThT) fluorescence. Responsible diagnostic laboratories report detection sensitivities for sCJD between 87 and 96% and specificities between 98 and 100%.^6^, ^7^, ^8^, ^9^ More specifically, the Canadian laboratory, funded by the Public Health Agency of Canada, reports a sensitivity of 95% and a specificity of 99%.^10^, ^11^

Since its development in 2010, QuIC assay performance has improved through optimization of reaction conditions and rPrP substrates.^12^ Substrate selection remains the linchpin of the tests diagnostic sensitivity and specificity, requiring correctly folded rPrP that efficiently converts in the presence of prion seeds while remaining stable in their absence. Numerous mammalian, chimeric, truncated, and mutant forms have been evaluated.^6^, ^9^, ^13^, ^14^, ^15^, ^16^, ^17^ The adoption of a truncated hamster rPrP proved most impactful, enabling higher reaction temperatures, faster run times, and greater sensitivity, leading to the second‐generation real‐time (RT)‐QuIC (IQ‐CSF RT‐QuIC).^18^, ^19^

Our laboratory developed an adaptation of QuIC for Canadian diagnostics, end‐point QuIC (EP‐QuIC), using full‐length hamster rPrP and a mechanical shaker with enhanced shearing capacity. EP‐QuIC achieves performance comparable to or exceeding IQ‐CSF RT‐QuIC, with nearly 3,000 CSF samples tested with this method in Canada since 2016 (Fig S1).^10^, ^11^ Despite the strong diagnostic performance of EP‐QuIC, the test fails to detect approximately 4 to 10% of sCJD cases and some IPDs. Similar to other QuIC tests, sensitivity is particularly low for the rare VV1 and MM2 sCJD subtypes, VPSPr, fatal familial insomnia (FFI), and Gerstmann‐Sträussler‐Sheinker disease (GSS).^18^, ^20^, ^21^, ^22^, ^23^

In this study, we evaluated a novel rPrP substrate derived from the deer mouse (*Peromyscus maniculatus*) sequence for QuIC‐based diagnostic testing of CSF. This species, previously used in our laboratory to model sCJD and chronic wasting disease (CWD),^24^, ^25^ expresses PrP that differs from bank vole PrP by only 2 residues (D227/R230 in deer mouse vs E227/S230 in bank vole) at a critical region for PrP^C^ to PrP^Sc^ conversion.^26^, ^27^, ^28^ Using this substrate, we achieved universal detection of sCJD cases and observed a marked improvement in identifying IPDs and VPSPr cases that generally yield negative or indeterminate results with RT‐QuIC, IQ‐RT‐QuIC and EP‐QuIC.

## Methods

### Samples and Ethics

CSF samples were collected in accordance with our diagnostic program from symptomatic Creutzfeldt‐Jakob disease (CJD) and or non‐CJD patients and stored at −80°C. Additional CSF samples were obtained from the National Prion Disease Pathology Surveillance Center in the United States. Brain tissue was collected through autopsy in accordance with our surveillance program for probable CJD cases and stored at −80°C. This study involves the secondary use of brain and CSF tissues. All patient tissues were obtained with the necessary consent for research use, as approved by the Health Canada‐Public Health Agency of Canada Research Ethics Board (REB reference numbers 2009‐036P and 2017‐009P). Demographic information available to the Canadian diagnostic laboratory is limited to the information provided at the time of sample testing (patient age, gender). Additional information such as symptoms, disease durations, and results from other clinical investigations are not routinely made available to the diagnostic lab and were, therefore, excluded from the study. Infectious work was performed under biosafety level 2+ (BSL‐2+) conditions or higher.

### Substrate Preparation

Substrate was prepared as previously described,^29^ with modifications. rPrP was produced in‐house using either the full‐length (amino acids 23–231) or truncated version (amino acids 90–231) of the Syrian Golden Hamster PRNP (prion encoding gene) open reading frame (ORF) (GenBank accession no. XP_012967855), or the full‐length (amino acids 23–230) deer mouse *PRNP* ORF (GenBank accession no. ACV85683), the full‐length (amino acids 23–231) bank vole *PRNP* ORF (GenBank accession no. XTO94710), or the human (amino acids 23–231) *PRNP* ORF (GenBank accession no. BCK59655.1). Site‐directed mutagenesis using the Q5 Site‐Directed Mutagenesis Kit (New England Biolabs, Ipswitch, MA) was used to generate the Human E200K‐variant *PRNP* ORF plasmid. The genes were cloned into vector pET41a, expressed in *E. coli* Rosetta cells and grown in Luria‐Bertani (LB) supplemented with kanamycin and chloramphenicol. Expression was induced using either 1mM isopropyl‐β‐D‐thiogalactopyranoside (IPTG) (Millipore Sigma, Burlington, MA) or the Overnight Express Autoinduction System 1 (Millipore Sigma) at 37°C for 28 hours. Cells were pelleted by centrifugation and homogenized using BugBuster Master Mix (Millipore Sigma) to isolate rPrP inclusion bodies. Inclusion bodies were denatured in 8M guanidine hydrochloride with 0.1M sodium phosphate, pH 8.0, and denatured protein was incubated with 18 to 54g of NiNTA Superflow resin (Qiagen, Hilden, Germany) with end‐over‐end mixing at room temperature. Loaded resin was packed into an XK 26/20 column (Cytiva, Marlborough, MA) and equilibrated over 1.5 column bed volumes with denaturing buffer (0.2M sodium phosphate monobasic, 0.2M sodium phosphate dibasic, 10mM tris, 6M guanidine hydrochloride; buffered to pH 8.0 with hydrochloric acid (HCl)) at a flow rate of 2.5mL/min using an AKTA Pure 25 chromatography system (GE Healthcare, Chicago, IL). On‐column refolding was performed by applying a linear gradient of refolding buffer (0.2M sodium phosphate dibasic, 0.2M sodium phosphate monobasic, 10mM Tris; buffered to pH 5.8 with HCl) at a flow rate of 0.0417mL/min per gram of resin over 240 minutes. The substrate was eluted in refolding buffer supplemented with 500mM imidazole at a flow rate of 0.111mL/min per gram of resin. A total of 2mL fractions were collected into 1.3mL ice cold dialysis buffer (0.2M sodium phosphate dibasic, 0.2M sodium phosphate monobasic; buffered to pH 5.8 with HCl) using an AKTA F9‐C fraction collector. Pooled fractions were immediately dialyzed for 22 hours against 2L dialysis buffer, with 1 buffer exchange the following day before storing at −80°C. Protein concentration was determined by NanoDrop using molar extinction coefficients of 62,005M^−1^ cm^−1^ (full‐length Syrian golden hamster), 26,025M^−1^ cm^−1^ (Truncated Syrian golden hamster) or 61,880M^−1^ cm^−1^ (full‐length deer mouse).

### Tissue Preparation

A total of 100mg of cortex brain tissue was placed in a biomasher filter tube (OMNI International, Kennesaw, GA) and homogenized using a disposable homogenizer column and pestle. The column was centrifuged at 14,500rpm for 30 seconds to filter out solid particulates. Following the removal of the filter of the column, 900μL of Dulbecco's phosphate‐buffered saline (DPBS) was added to the tissue to yield a 10% brain homogenate. The homogenate was vortexed for 1 minute and centrifuged at 15,000 × *g* for 10 minutes. The supernatant was removed and stored at −80°C.

### RT‐QuIC Using CSF and Brain Tissue

RT‐QuIC using CSF was performed as previously described,^8^, ^9^ with modifications. CSF was aliquoted to ensure identical freeze–thaw cycles across all tests. Briefly, 15μL of CSF was added in triplicate to a reaction buffer containing 10μg rPrP, 300mM sodium chloride (NaCl), 1mM ethylenediaminetetraacetic acid (ETDA), and 10μM thioflavin T (ThT) in phosphate‐buffered saline (PBS) to a final volume of 100μL. For reactions using truncated hamster substrate (Ha rPrP 90‐231), a final concentration of 0.002% (w/v) sodium dodecyl sulfate (SDS) was added to the reaction mixture. Plates were incubated at 42°C or 55°C for Ha rPrP 90‐231, in a FLUOstar Omega microplate reader (BMG Labtech, Ortenberg, Germany). ThT fluorescence was measured every 15 minutes for 100 hours (or 60 hours for Ha rPrP 90‐231) during cycles of double‐orbital shaking at 700rpm for 60 seconds followed by 60 seconds of rest. Samples were considered positive if the fluorescence signal crossed a threshold defined as 5 times the standard deviation plus the mean of all fluorescence values recorded on the plate between 30 and 90 minutes. For deer mouse substrate (DM rPrP 23‐230), the positivity threshold was set at 20 times the standard deviation plus the mean fluorescence during the same time interval. RT‐QuIC using brain tissue followed the same protocol with modifications. Ten percent (w/v) brain homogenate was serially diluted 10‐fold in PBS supplemented with 0.1% (w/v) SDS and 1% (w/v) N2 supplement (Gibco, Grand Island, NY). Next, 2.1μL of diluted brain homogenate was combined with 10μg rPrP in reaction buffer (final concentration of 1mM EDTA, 10μM ThT, and 0.002% [w/v] SDS). All samples were run in quadruplicate. Each plate included positive controls consisting of 10% brain homogenate from hamster 263 K prion‐infected tissue and negative controls from PBS (mock)‐infected hamsters.

### EP‐QuIC Using CSF

EP‐QuIC using CSF was performed as previously described.^30^ Briefly, 15μL of CSF was added in triplicate to a reaction buffer containing 10μg rPrP, 160mM NaCl, 10μM EDTA, and 10μM ThT in PBS, to a final volume of 100μL. Plates were incubated in an Eppendorf Thermomixer C at 42°C for 20 minutes with intermittent shaking cycles of 900rpm (90‐second shaking followed by 30 seconds rest). A bottom‐read fluorescence intensity measurement was recorded and expressed as relative fluorescence units. Following the initial read, incubation continued for 66 hours for full‐length hamster rPrP and 50 to 66 hours for deer mouse rPrP under the same shaking conditions on the Thermomixer. A final fluorescence intensity reading was taken at the end of the respective incubation periods. Samples with 2 or more replicates exhibiting at least a 4‐fold increase in fluorescence over the initial reading were considered positive. Samples with only 1 positive replicate were re‐run, and if the result was reproducible with a single positive replicate, the sample was classified as indeterminate. Sensitivity and specificity with 95% confidence intervals were calculated using the Wilson score method.

### Statistical Analysis

Diagnostic sensitivity and specificity with 95% confidence intervals were calculated using the Wilson score method. Differences in the proportion of positive and negative QuIC tests across substrates were evaluated using the 2‐sided Fisher's exact test, chosen because of the small sample sizes. Pairwise comparisons (deer mouse (DM) vs. full‐length hamster(FL), DM vs. truncated hamster (Tr), FL vs Tr) was performed, with significance defined as *p* < 0.05. Groups showing significant differences are indicated in the main tables, and full *p*‐values are provided in the results. Statistical analyses were performed using GraphPad Prism (version 10.6.1).

## Results

### Optimization of EP‐QuIC Using Deer Mouse PrP Substrate

**FIGURE 1:**
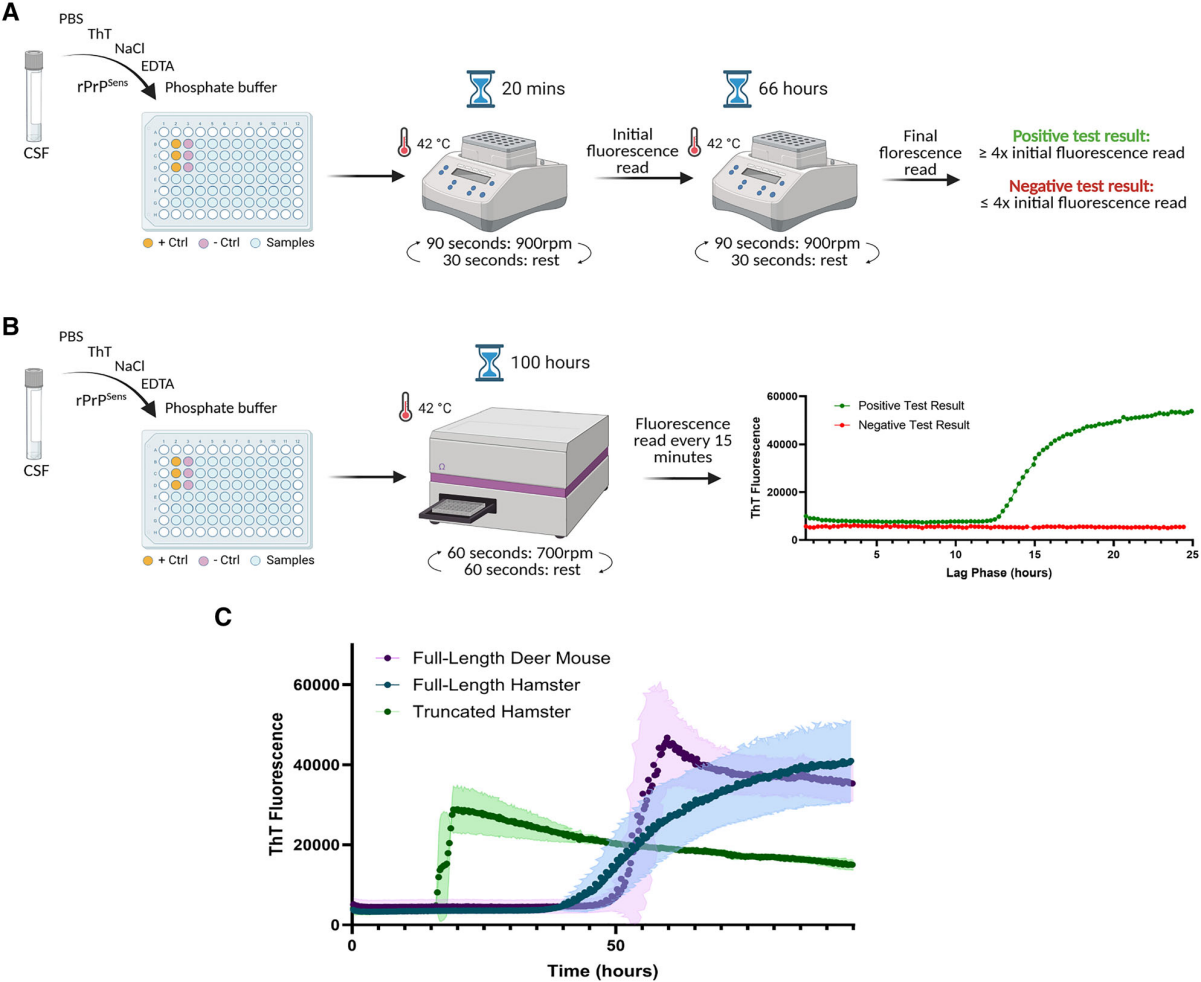
Comparison of end‐point (EP)‐ and real‐time (RT) quaking‐induced conversion (QuIC) methodologies, along with substrate‐dependent RT‐QuIC analysis. Schematic representation of the Canadian diagnostic workflow for (A) for EP‐QuIC and (B) RT‐QuIC. (C) Grouped representative RT‐QuIC data from cerebrospinal fluid (CSF) of confirmed Creutzfeldt‐Jakob disease (CJD) patients (n = 5) with full‐length deer mouse, full‐length hamster, and truncated hamster substrates. Data is shown as the mean ± standard deviation for each time point (shown as shaded area around curve) of Thioflavin T (ThT) fluorescence.

Since 2016, the Canadian CJD diagnostics and reference laboratory has used EP‐QuIC as an accredited CSF test (Fig 1A). Seeding is initiated with 15μL of CSF in a standard reaction mix, but performed on an Eppendorf Thermomixer at 42°C with circular shaking cycles (90 seconds at 900rpm, 30 seconds rest), rather than on the Fluorstar Omega (see Fig 1B). As no amplification trace is generated, a test is deemed positive if the fluorescence difference between the 20‐minute baseline and the 66‐hour end‐point exceeds a predefined threshold optimized using the full‐length hamster substrate (FL‐rPrP). Because seeding dynamics vary by rPrP sequence, we first optimized these criteria for the deer mouse substrate (DM‐rPrP), which similar to FL‐rPrP, showed a distinct fluorescent signal peaking between 50 and 66 hours (see Fig 1C). A fluorescent reading was recorded at both 50 and 66 hours during test development. We used a positivity threshold to a fluorescence signal exceeding the main baseline plus 20 standard deviations for DM‐rPrP.

### RT‐QuIC and EP‐QuIC Assays Using DM‐rPrP Seeded by sCJD CSF Specimens

We conducted a direct comparative analysis FL‐rPrP and DM‐rPrP substrates in CSF‐seeded EP‐QuIC assays from individuals diagnosed with definite or probable sCJD. In total, 61 CSF samples submitted for diagnostic testing between 2014 and 2024 were included in the panel (Table S1). All sCJD samples tested positive using the DM‐rPrP substrate (Table 1). CSF from 71 non‐CJD cases (pathologically confirmed) were also tested and yielded negative results, confirming comparable sensitivity and specificity between substrates.

**TABLE 1:** Comparative Performance of PrP Substrates in CSF RT‐ and EP‐QuIC Assays for sCJD and Non‐CJD Cases

Test	Substrate	No. of cases						Sensitivity (%) (95% CI)	Specificity (%) (95% CI)
		sCJD			Non‐CJD				
		TP	IND	FN	TN	IND	FP		
EP‐QuIC	Full‐length hamster	61	0	0	71	0	0	100 (94.1–100.0)	100 (94.1–100.0)
	Deer mouse	61	0	0	71	0	0	100 (94.1–100.0)	100 (94.1–100.0)
RT‐QuIC	Full‐length hamster	51	8	2	69	1	1	83.6 (72.4–90.8)	97.1 (90.3–99.2)
	Deer mouse	51	7	3	69	1	1	83.6 (72.4–90.8)	97.1 (90.3–99.2)
	Truncated hamster	58	0	3	68	1	2	95.1 (86.5–98.3)	95.8 (88.3–98.6)

CI = confidence interval; CJD = Creutzfeldt‐Jakob disease; CSF = cerebrospinal fluid; EP = end‐point; FN = false negative (0/3 wells positive); FP = false positive (3/3 wells positive); IND = indeterminate (1/3 wells positive); PrP = prion proteins; QuIC = quaking‐induced conversion; RT = real‐time; sCJD = sporadic Creutzfeldt‐Jakob disease; TN = true negative (0/3 wells positive); TP = true positive (2–3 wells positive).

As RT‐QuIC and IQ‐CSF RT‐QuIC are the diagnostic assays most commonly used in laboratories worldwide to test for seeding in suspected cases, we present data for RT‐QuIC analysis using both FL‐rPrP and DM‐rPrP for all 61 CJD cases and non‐CJD cases (75 tested with FL‐rPrP and 35 tested with DM‐rPrP), alongside IQ‐CSF‐RT‐QuIC using the truncated hamster (T‐rPrP) substrate (Table 1). DM‐rPrP and FL‐rPrP substrates yielded comparable results in RT‐QuIC, while IQ‐CSF RT‐QuIC using T‐rPrP demonstrated higher sensitivity, which is in line with previous reports.^9^, ^18^, ^19^, ^31^

**FIGURE 2:**
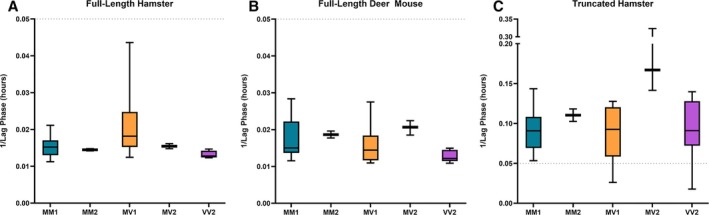
Seeding profiles of cerebrospinal fluid (CSF) from positive sporadic Creutzfeldt‐Jakob disease (sCJD) cases in real‐time (RT)‐quaking‐induced conversion (QuIC) using full‐length hamster, full‐length deer mouse, and truncated hamster prion protein substrates. CSF samples from sCJD subtypes MM1 (n = 26), MM2 (n = 2), MV1 (n = 7), MV2 (n = 3), and VV2 (n = 10) were tested by RT‐QuIC using the following substrates: (A) full‐length hamster prion proteins (PrP) (amino acids 23‐231), (B) deer mouse PrP (amino acids 23‐230), and (C) truncated hamster PrP (amino acids 90‐231). The fluorescent threshold used to determine the lag phase was defined as the signal exceeding 5 times the standard deviation plus the average fluorescence of all wells on the plate during the baseline period (30–90 minutes).

In Figure 2, we provide data to show the lag phases for each subtype represented among 48 positive cases in our cohort. These included 26 MM1, 2 MM2, 7 MV1, 3 MV2, and 10 VV2 cases. The lag phases determined from RT‐QuIC traces showed similar trends across sCJD subtypes for both FL‐rPrP and DM‐rPrP substrates. The T‐rPrP substrate demonstrated shorter incubation times, but increased variability in lag phases within each sCJD subtype, as well as markedly longer lag phases for the 3 MV2 cases. We observed that slow lag phases correlated with lowered sensitivity. Substrates with prolonged lag times generally required higher concentrations of seeding activity to generate a detectable signal, resulting in higher limits of detection.

### A Comparative Analysis of RT‐QuIC Assay Performance Using Brain Homogenate Identifies Deer Mouse Substrate as the most Sensitive for the Detection of Rare and Genetic Prion Disease Variants

To conserve CSF from rare cases, we next compared substrate performance in RT‐QuIC assays seeded with a dilution series of brain homogenates from representative Canadian CJD cases. Although brain homogenate contains markedly higher concentrations of PrP^Sc^ compared to CSF, substrate‐specific differences in detection sensitivity appear to be conserved across tissues. Therefore, using brain homogenate enables a reliable assessment of relative substrate performance and provides insight translatable to CSF. This approach is advantageous when evaluating seeding characteristics in QuIC as semi‐quantitative, robust data is produced for direct comparisons of seeding under identical conditions. Brain homogenates from common sCJD subtypes (MM1, MV2, and VV2) contain seeding activity that is readily detected by standard substrates, even in dilutions down to 10^−8^. We estimate that the concentration of prions in CSF is equivalent to brain homogenate diluted 10^−6^ or below (data not shown). Preliminary unpublished data from our lab suggest that seeding activity from sCJD MM1, MV2, and VV2 homogenates might be uniformly detected by all substrates. Contrary to this, seeding activity from rare sCJD subtypes and IPD cases is more challenging to detect, even in brain homogenates. Therefore, we focused on these cases to further compare the performance of deer mouse substrate in RT‐QuIC with previously described substrates.

**FIGURE 3:**
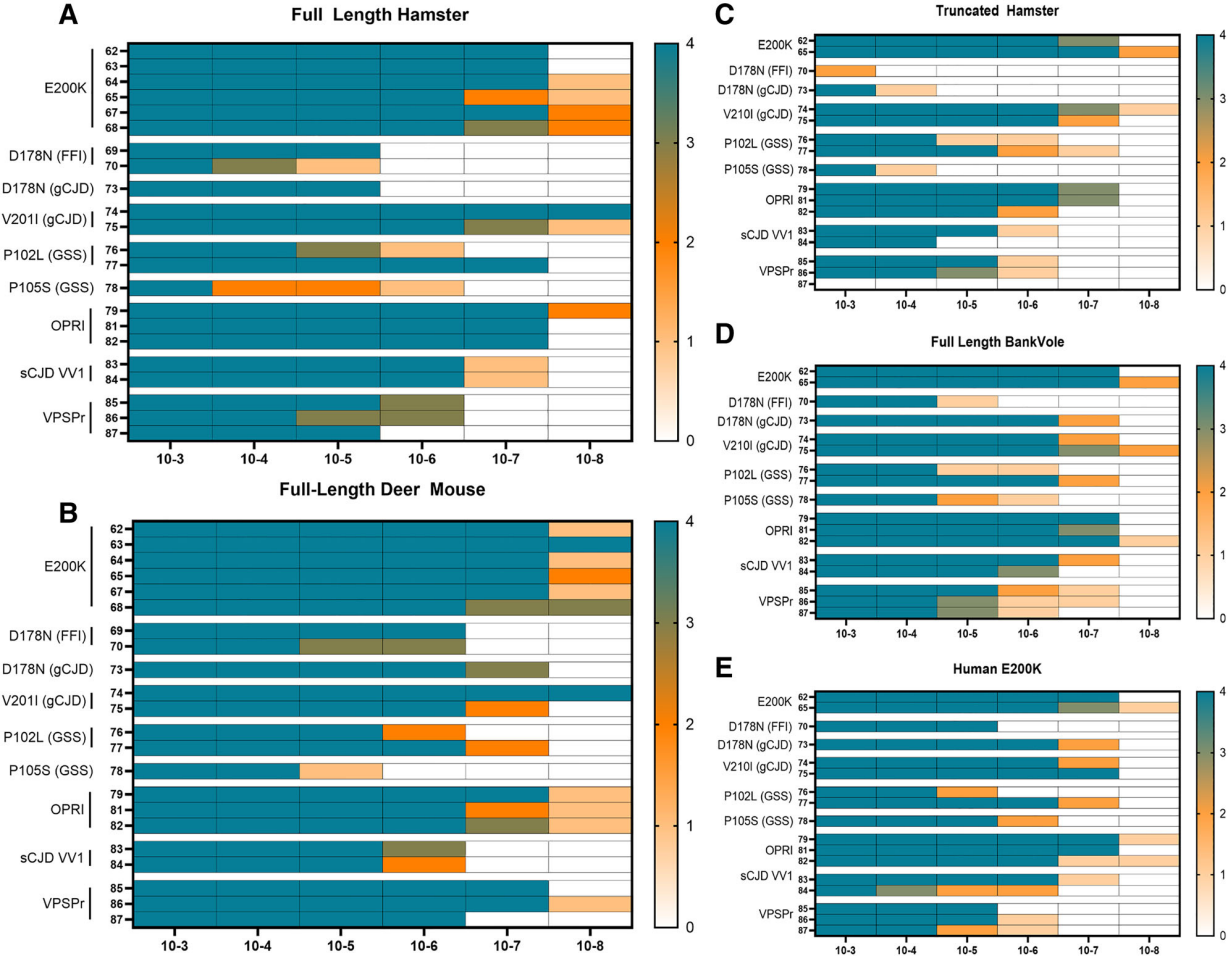
Real‐time (RT) quaking‐induced conversion (QuIC) seeding activity from brain homogenate from inherited and rare sporadic prion disease cases using various recombinant prion proteins (PrP) substrates: full‐length hamster, full‐length deer mouse, truncated hamster, bank vole, and human E200K. Brain homogenates (10% w/v) were serially diluted to 10^−^8 and tested in quadruplicate using RT‐QuIC. Heat maps depict the number of positive wells at each dilution for each sample using (A) full‐length hamster, (B) full‐length deer mouse, (C) truncated hamster, (D) full‐length bank vole, and (E) human E200K rPrP.

We selected brain homogenate from various IPDs and rare sCJD subtypes known to exhibit reduced seeding activity in CSF RT‐QuIC to evaluate DM‐rPrP as a universal substrate. Figure 3 compares substrates in RT‐QuIC reactions seeded with brain homogenate dilution series from cases of E200K, FFI, D178N gCJD, GSS, V210I, OPRI (2‐ and 5‐OPRI), and sCJD including the rare VV1 subtype as well as VPSPr. We used the FL‐rPrP, DM‐rPrP, and T‐rPrP substrates as well as recombinant bank vole (BV‐rPrP) and a human E200K mutation (E200K‐rPrP) for genetic and atypical sCJD cases. Seeding results are color coded according to RT‐QuIC positivity across replicates to determine the minimum brain homogenate dilution with detectable seeding activity, referred to here as the detection limit.

We observed substrate‐dependent detection limits across IPDs and rarer sCJD subtypes. For example, the DM‐rPrP substrate showed superior or equivalent sensitivity of detection to FL‐rPrP in nearly all CJD and IPD types. By contrast, T‐rPrP was particularly insensitive to some of the IPD types, most strikingly those with the D178N and GSS mutations. This was also true for the sCJD subtype VV1 and VPSPr. These patterns align with testing performed at various international testing centers, where the sensitivity to detect D178N, GSS, VPSPr, and VV1 are found to be particularly low. Although a human backbone of the E200K‐rPrP substrate has been proposed to improve detection in selected IPDs, it did not outperform DM‐ or FL‐rPrP in this study.

**FIGURE 4:**
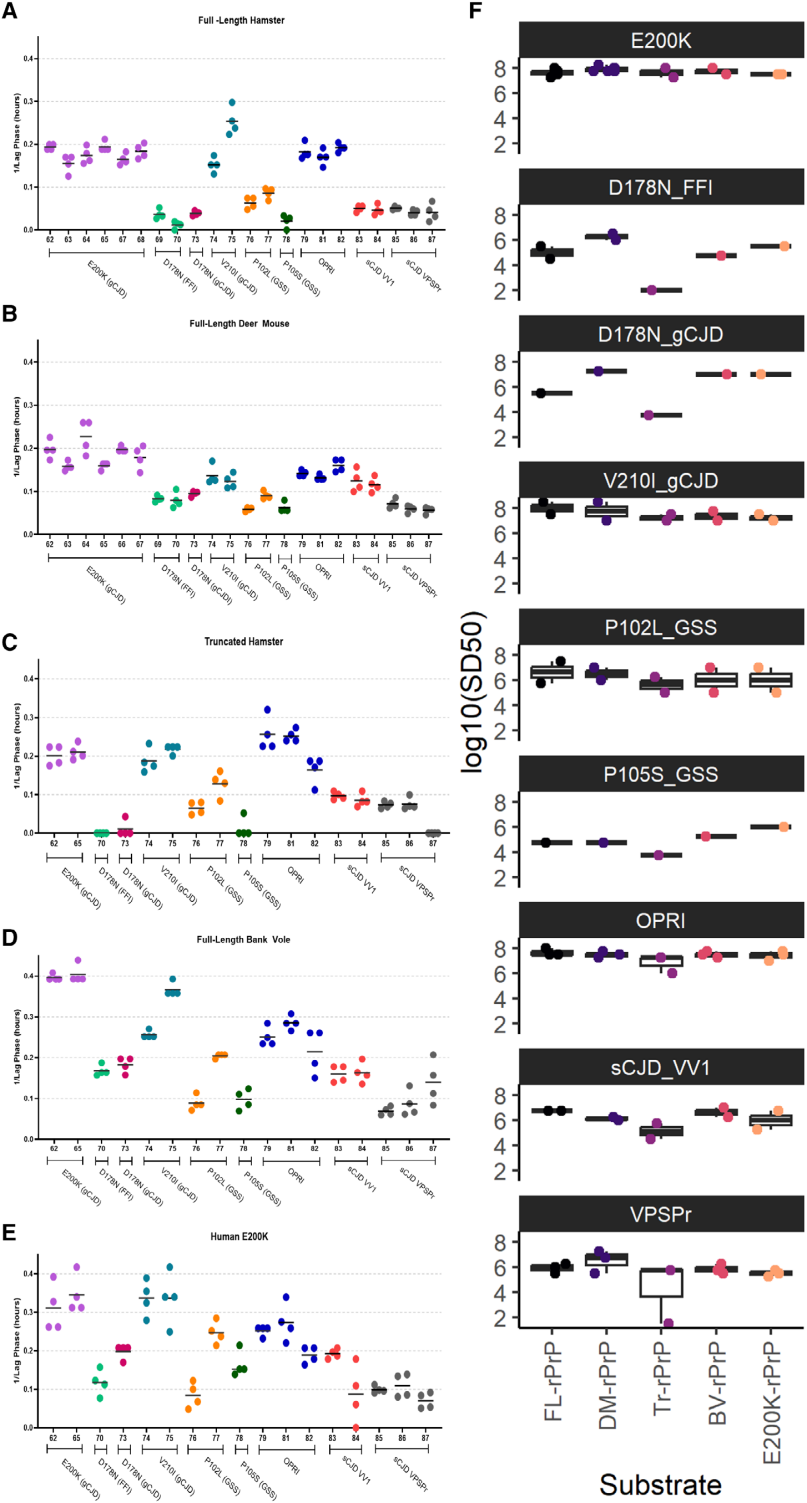
Real‐time (RT) quaking‐induced conversion (QuIC) lag phases and seeding doses across recombinant prion proteins (PrP) substrates for sporadic Creutzfeldt‐Jakob disease (sCJD) and inherited prion disease cases. Lag phases of RT‐QuIC seeding activity using 10% (w/v) brain homogenate at the 10^−4^ dilution (4 technical replicates per sample) are shown for each sample using (A) full‐length hamster rPrP, (B) full‐length deer mouse rPrP, (C) truncated hamster rPrP, (D) full‐length bank vole rPrP, and (E) human E200K rPrP. (F) Boxplots depict the 50% seeding dose, or SD_50_, calculated for each recombinant PrP substrate, grouped by inherited prion disease and sCJD subtype

We also compared the lag phases in each RT‐QuIC reaction seeded by 2μL of brain homogenate from each case at a single dilution, 10^−4^ (Fig 4). We found that the relative variations in lag phase in each CJD subtype or IPD followed similar trends across all substrates. A notable difference was the consistently longer lag phase for the D178N mutations in both substrates with the hamster sequence backbone, either FL‐rPrP or T‐rPrP, versus the other substrates tested. Overall, the lag phases of DM‐rPrP in each of the cases showed consistently low variability and high consistency across the samples tested.

### Deer Mouse Substrate Enables Detection of Prions in Several Inherited Prion Disorders That Are Refractory to Diagnosis by EP‐QuIC Assay

Given the superior performance of the DM‐rPrP substrate in comparative assays seeded with brain homogenate, we validated its performance in a cohort of 54 IPD, rare CJD subtypes, and VPSPr cases provided from our Canadian CJD Diagnostics program and the United States (US) National Prion Disease Pathology Surveillance Center. We performed EP‐QuIC tests using both DM‐rPrP and FL‐rPrP. In addition, all samples were tested by second generation RT‐QuIC (IQ‐RT‐QuIC). Details of CSF samples used are provided in Table S1 and the results of each test are summarized in Table 2. Comprehensive test results for each individual sample are also provided as Table S2.

**TABLE 2:** Comparative Performance of PrP Substrates in CSF RT‐ and EP‐QuIC for Inherited Prion Diseases and Atypical sCJD Subtypes

Diagnosis group	Subtype	Deer Mouse EP‐QuIC		FL. hamster EP‐QuIC		Tr. hamster RT‐QuIC	
		Pos.	Ind.	Pos.	Ind.	Pos.	Ind.
gCJD	E200K	7/7	–	7/7	–	7/7	–
	V210I	2/2	–	2/2	–	2/2^a^	–
	V180I	0/1	–	0/1	–	1/1^a^	–
	D178N	1/1	–	1/1^a^	–	0/1	–
	OPRI	4/4	–	4/4	–	4/4	–
FFI^a^	D178N	12/13^b^	1/13	4/13^b^	1/13	0/13	–
GSS^a^	P102L	10/10	–	8/10	–	4/10	–
	P105S	1/1	–	1/1^a^	–	0/1	–
	A117V	1/1	–	0/1	–	0/1	–
Atypical sCJD	VV1	5/5	–	5/5	–	2/5^a^	1/5
	MM2	5/5	–	5/5	–	3/5	2/5
	VPSPr	3/5	1/5	3/5	–	2/5	1/5

^a^ Indicates a significant difference between substrates for this diagnostic group (see Table S3.)

^b^ Indicates a positive results based on a 2/3 well positivity.

CJD = Creutzfeldt‐Jakob disease; CSF = cerebrospinal fluid; EP = end‐point; FFI = fatal familial insomnia; FL. = full‐length; GSS = Gerstmann‐Sträussler‐Sheinker disease; Ind = indeterminate (1/3 wells positive); Pos = positive result (2–3/3 wells positive); PrP = prion proteins; QuIC = quaking‐induced conversion; RT = real‐time; sCJD = sporadic Creutzfeldt‐Jakob disease; Tr. = truncated; VPSPr = variably protease‐sensitive prionopathy.

Although EP‐QuIC performed better than IQ‐RT‐QuIC in all cases tested, the inclusion of the DM‐rPrP substrate provided the most striking improvement in diagnostic accuracy. The D178N mutation associated with FFI is particularly refractory to testing by IQ‐RT‐QuIC, with all 13 cases tested producing negative results. EP‐QuIC with FL‐rPrP substrate detected 4 of 13 cases with 1 indeterminate test, while the DM‐rPrP substrate detected 12 of 13 cases with the remainder providing an indeterminate result. Similarly, all 12 GSS causative mutations resulted in positive tests with DM‐rPrP substrate, versus 4 of 12 for IQ‐RT‐QuIC. Similar performance was noted for the atypical sCJD subtype VV1 and MM2, of which all 10 were picked up by EP‐QuIC using either substrate, while IQ‐RT‐QuIC picked up 2 of 5 VV1 case, with 1 of 5 indeterminate, and 3 of 5 MM2 cases. VPSPr cases showed the least improvement in performance with 3 of 5 positive and 1 of 5 indeterminate in EP‐QuIC compared to 2 of 5 cases positive in IQ‐RT‐QuIC. Interestingly, the only case picked up by IQ‐RT‐QuIC that was not detected by EP‐QuIC is the extremely rare, low penetrance mutation V180I that shares some features of VPSPr proteinase resistant core.^32^

## Discussion

CSF QuIC seeding assays have become the gold standard for the laboratory diagnosis of CJD worldwide. Our laboratory uses a modified version of the standard methodology typically used by most diagnostic centers performing CJD testing, which has shown improved diagnostic utility over IQ‐RT‐QuIC. Surveillance data generated by our lab for the period 2016 to 2024 demonstrated a sensitivity of 95 to 96% and specificity of 99% for our EP‐QuIC test,^10^ comparable or higher than data from surveillance centers using second generation IQ‐RT‐QuIC.^9^, ^31^, ^33^, ^34^ Despite these impressive statistics, QuIC tests show lower sensitivity for some IPDs, particularly those with non‐CJD phenotypes characterized by prion protein amyloidosis (eg, GSS), and based on published reports, some atypical subtypes such as the rare VV1 type. These sensitivity limitations highlight a need for continued refinement of diagnostic assay protocols to ensure reliable antemortem CSF detection across the full spectrum of prion disease phenotypes. This is particularly important in light of the shift toward laboratory surveillance for CJD, driven by reductions in many national autopsy CJD surveillance programs.

By adapting EP‐QuIC to use the DM‐rPrP substrate in place of FL‐rPrP, we maintain high sensitivity and specificity for detecting sCJD while expanding the assay's ability to reliably detect previously refractory cases of IPD and atypical subtypes, as validated in a cohort of Canadian and US cases. We also report a convenient method to evaluate substrate seeding efficacy, by comparing the limit of detection for various substrates under identical assay conditions using a dilution series of brain homogenate. This allows a semi‐quantitative framework for directly assessing test parameters while conserving limited amounts of CSF of rare cases for use in proficiency panels and validation. We note that relative seeding efficiency assessed by limiting dilution directly mirrored testing the detection performance shown by each substrate on validation in CSF panels. One of the most intriguing findings of this analysis was the poor performance of T‐rPrP in amplifying some IPD seeds. This was particularly evident for the D178N mutations that are found in FFI (when cis‐129 is methionine) or gCJD (when cis‐129 is valine) and corroborating the poor performance of second‐generation IQ‐RT‐QuIC in diagnostic testing of individuals with these mutations. Similar insensitivity was observed in GSS cases, which were consistently detected by EP‐QuIC with DM‐rPrP, with all tested cases successfully identified. Efficient seeding of the VV1 subtype also translated to 100% detection of these cases in EP‐QuIC versus lower sensitivity shown by IQ‐RT‐QuIC. Given the widespread diagnostic application of T‐rPrP in RT‐QuIC, we also conducted a brief evaluation of this substrate under EP‐QuIC conditions (data not shown). Although approximately 50% of wells showed positive responses, we also observed a high rate of false positives among negative CSFs tested in parallel. Although further optimization may improve performance, its current limitations are substantial enough that it is not a viable candidate for diagnostic consideration at this time.

It is interesting to postulate on the features of the primary sequence of deer mouse prion protein that led it to efficiently amplify multiple prion strains and genetic variants. Although our work with the deer mouse began fortuitously, as a colony is maintained at our institute, prior studies have shown that substituting the bank vole residues E227/S230 with D227/R230 yields a PrP sequence highly permissive to cross‐species transmission, highlighting the functional significance of this C‐terminal region.^27^ The resulting D227/R230 sequence is identical to that of the deer mouse *PRNP* gene. The extreme C terminus of the prion protein appears to be a key element controlling the conversion of PrP^C^ to PrP^Sc^, with residues 227 and 230 (bank vole numbering) particularly important in promoting efficient templated misfolding.^26^, ^27^, ^28^ We hypothesize that in this context, the deer mouse protein retains its sensitivity for fibril replication and growth as found for the bank vole protein, while the presence of the mouse residues D227/R230 increases stability, so reducing the spontaneous amyloid formation observed when bank vole prion protein is used as RT‐QuIC substrate, therefore, maintaining exceptional specificity. How this stabilizing effect might relate to cross‐species transmission remains an avenue for future study.

The EP‐QuIC test with DM‐rPrP substrate, termed enhanced sensitivity QuIC or ES‐QuIC, represents an improvement in the diagnostic utility of quaking induced conversion for the laboratory diagnosis of human prion diseases. EP‐ and ES‐QuIC also offer the advantage of removing the subjectivity of interpreting an RT‐QuIC trace to a simple algorithm applied to the ratio of initial/final read fluorescence. The improved sensitivity and specificity achieved with the ES‐QuIC assay also raises important considerations for future clinical applications. In particular, its performance suggests potential utility in settings where longitudinal monitoring of prion seeding activity may become clinically informative. Although prospective serial CSF samples were not available in this study, the reduced spontaneous amyloid formation and consistent performance across sporadic, inherited, and atypical prion diseases indicate this test may be well suited for future clinical trials evaluating disease progression or therapeutic response, especially in genetically at‐risk individuals or in early‐stage disease. As therapeutics for inherited prion diseases advance toward clinical testing, the ability to sensitively detect changes in prion seeding activity over time may become increasingly important, and our ES‐QuIC test represents a promising candidate for such applications. Prospective longitudinal validation will, however, be required to establish its performance in monitoring prion seeding activity over time.

The EP‐QuIC test used in our laboratory has detected only 1 validated false positive test result over the past 8 years based on evaluation of autopsy confirmed cases.^10^ Although we are just beginning to use the DM‐rPrP substrate routinely for CJD diagnostic testing in Canada, experimental data so far shows no reduction in specificity when compared with FL‐rPrP. Broader clinical implementation, however, will require validation in independent laboratories, assessment of inter‐laboratory reproducibility, protocol harmonization, and consideration of practical factors related to production and standardization of deer mouse substrate. We view EP‐QuIC as a highly sensitive test that should replace RT‐QuIC and IQ‐RT‐QuIC in the diagnostic laboratory given its proven reproducibility shown in our laboratory over the past 8 years.^10^ Following validation of the DM‐rPrP substrate in extended use alongside FL‐rPrP, its utility will enable laboratory testing of CSF to reliably be used to identify human prion disease antemortem in any case of neuropsychiatric syndrome lacking an alternative diagnosis. Its utility in cases where current test sensitivity is low, such as FFI, GSS, and the VV1 sCJD subtype cases, reliance on autopsy for confirmatory diagnosis of these cases will be reduced. However, autopsy and neuropathologic examination of brain tissue should remain an important part of CJD surveillance to confirm disease etiology and to identify novel forms of prion disease potentially related to iatrogenic or zoonotic exposure. This is particularly important in North America where CWD in deer is endemic and may pose a risk of transmission to humans.

## Author Contributions

J.M., R.F., D.M.S.K., B.A.B.E., and S.A.B contributed to the conception and design of the study; J.M., R.F., D.M.S.K., L.L., M.L., K.A., C.P., K.F., O.N., J.S., S.S., B.S.A., and S.A.B contributed to the acquisition and analysis of data; J.M., R.F., D.M.S.K., B.A.B.E., J.S., and S.A.B contributed to drafting the test or preparing the figures.

## Potential Conflicts of Interest

Nothing to report.

## Supporting information


**Figure S1.** End Point Quaking‐Induced Conversion (EP‐QuIC) assay diagnostic testing process for suspected prion diseases cases in Canada (2014–2024).
**Table S1.** Study Cohort Description.
**Table S2.** Individual QuIC test results for inherited prion disease and rare sCJD subtype cases, using various substrates for EP‐QuIC, IQ‐QuIC, and RT‐QuIC.
**Table S3.** Statistical Analysis of CSF Substrate Performance in Genetic and Atypical Prion Diseases.

## Data Availability

All data generated or analyzed during this study are included in this article.
