# Supplementary material for: Enhanced Sensitivity of a Modified Quaking‐Induced Conversion Diagnostic Test for the Broad Detection of Sporadic and Inherited Prion Diseases: A Retrospective Study
Source: Ann Neurol. 2026 Jan 26;99(5):1303–14. doi: 10.1002/ana.78162 (PMC13092778; doi:10.1002/ana.78162)
Supplement: Supplementary file 1 — Figure S1. End Point Quaking‐Induced Conversion (EP‐QuIC) assay diagnostic testing process for suspected prion diseases cases in Canada (2014–2024). Table S1. Study Cohort Description. Table S2. Individual QuIC test results for inherited prion disease and rare sCJD subtype cases, using various substrates for EP‐QuIC, IQ‐QuIC, and RT‐QuIC. Table S3. Statistical Analysis of CSF Substrate Performance in Genetic and Atypical Prion Diseases. [file ANA-99-1303-s001.docx]

**Supplementary Appendix:**

| **Section** | **Page** |
| --- | --- |
| Methods: |  |
| - *Supplemental Figure 1. End Point Quaking-Induced Conversion (EP-QuIC) assay diagnostic testing process for suspected prion diseases cases in Canada (2014-2024)* | *2* |
| - *Supplemental Table 1. Study Cohort Description* | *3-6* |
| Results: |  |
| - *Supplemental Table 2. Individual QuIC test results for inherited prion disease and rare sCJD subtype cases, using various substrates for EP-QuIC, IQ RT-QuIC, and RT-QuIC.* | *7-8* |
| - *Supplemental Table 3. Statistical Analysis of CSF Substrate Performance in Genetic and Atypical Prion Diseases.* | *9* |

**Supplemental Figure 1.** End Point Quaking-Induced Conversion (EP-QuIC) assay diagnostic testing process for suspected prion disease cases in Canada (2014-2024).

Supplemental Table 1. Study Cohort Description

| **Case Number** | **Prion Disease Type** | **PRNP Mutation** | **Codon 129 Genotype** | **Biochemical Type** | **Country of Origin** | **Tissue Analysed** |
| --- | --- | --- | --- | --- | --- | --- |
| 1 | Definitive CJD | - | MM | Type 1 | Canada | CSF |
| 2 | Definitive CJD | - | MM | Type 1 | Canada | CSF |
| 3 | Definitive CJD | - | MM | Type 1 | Canada | CSF |
| 4 | Definitive CJD | - | MM | Type 1 | Canada | CSF |
| 5 | Definitive CJD | - | MM | Type 1 | Canada | CSF |
| 6 | Definitive CJD | - | MM | Type 1 | Canada | CSF |
| 7 | Definitive CJD | - | MM | Type 1 | Canada | CSF |
| 8 | Definitive CJD | - | MM | Type 1 | Canada | CSF |
| 9 | Definitive CJD | - | MM | Type 1 | Canada | CSF |
| 10 | Definitive CJD | - | MM | Type 1 | Canada | CSF |
| 11 | Definitive CJD | - | MM | Type 1 | Canada | CSF |
| 12 | Definitive CJD | - | MM | Type 1 | Canada | CSF |
| 13 | Definitive CJD | - | MM | Type 1 | Canada | CSF |
| 14 | Definitive CJD | - | MM | Type 1 | Canada | CSF |
| 15 | Definitive CJD | - | MM | Type 1 | Canada | CSF |
| 16 | Definitive CJD | - | MM | Type 1 | Canada | CSF |
| 17 | Definitive CJD | - | MM | Type 1 | Canada | CSF |
| 18 | Definitive CJD | - | MM | Type 1 | Canada | CSF |
| 19 | Definitive CJD | - | MM | Type 1 | Canada | CSF |
| 20 | Definitive CJD | - | MM | Type 1 | Canada | CSF |
| 21 | Definitive CJD | - | MM | Type 1 | Canada | CSF |
| 22 | Definitive CJD | - | MM | Type 1 | Canada | CSF |
| 23 | Definitive CJD | - | MM | Type 1 | Canada | CSF |
| 24 | Definitive CJD | - | MM | Type 1 | Canada | CSF |
| 25 | Definitive CJD | - | MM | Type 1 | Canada | CSF |
| 26 | Definitive CJD | - | MM | Type 1 | Canada | CSF |
| 27 | Definitive CJD | - | MM | Type 2 | Canada | CSF |
| 28 | Definitive CJD | - | MM | Type 2 | Canada | CSF |
| 29 | Definitive CJD | - | MV | Type 1 | Canada | CSF |
| 30 | Definitive CJD | - | MV | Type 1 | Canada | CSF |
| 31 | Definitive CJD | - | MV | Type 1 | Canada | CSF |
| 32 | Definitive CJD | - | MV | Type 1 | Canada | CSF |
| 33 | Definitive CJD | - | MV | Type 1 | Canada | CSF |
| 34 | Definitive CJD | - | MV | Type 1 | Canada | CSF |
| 35 | Definitive CJD | - | MV | Type 1 | Canada | CSF |
| 36 | Definitive CJD | - | MV | Type 2 | Canada | CSF |
| 37 | Definitive CJD | - | MV | Type 2 | Canada | CSF |
| 38 | Definitive CJD | - | MV | Type 2 | Canada | CSF |
| 39 | Definitive CJD | - | VV | Type 2 | Canada | CSF |
| 40 | Definitive CJD | - | VV | Type 2 | Canada | CSF |
| 41 | Definitive CJD | - | VV | Type 2 | Canada | CSF |
| 42 | Definitive CJD | - | VV | Type 2 | Canada | CSF |
| 43 | Definitive CJD | - | VV | Type 2 | Canada | CSF |
| 44 | Definitive CJD | - | VV | Type 2 | Canada | CSF |
| 45 | Definitive CJD | - | VV | Type 2 | Canada | CSF |
| 46 | Definitive CJD | - | VV | Type 2 | Canada | CSF |
| 47 | Definitive CJD | - | VV | Type 2 | Canada | CSF |
| 48 | Definitive CJD | - | VV | Type 2 | Canada | CSF |
| 49 | Probable sCJD | - | - | - | Canada | CSF |
| 50 | Probable sCJD | - | - | - | Canada | CSF |
| 51 | Probable sCJD | - | - | - | Canada | CSF |
| 52 | Probable sCJD | - | - | - | Canada | CSF |
| 53 | Probable sCJD | - | - | - | Canada | CSF |
| 54 | Probable sCJD | - | - | - | Canada | CSF |
| 55 | Probable sCJD | - | - | - | Canada | CSF |
| 56 | Probable sCJD | - | - | - | Canada | CSF |
| 57 | Probable sCJD | - | - | - | Canada | CSF |
| 58 | Probable sCJD | - | - | - | Canada | CSF |
| 59 | Probable sCJD | - | - | - | Canada | CSF |
| 60 | Probable sCJD | - | - | - | Canada | CSF |
| 61 | Probable sCJD | - | - | - | Canada | CSF |
| 62 | genetic CJD | E200K | MM | - | Canada | Brain |
| 63 | genetic CJD | E200K | MM | - | Canada | Brain & CSF |
| 64 | genetic CJD | E200K | MM | - | Canada | Brain & CSF |
| 65 | genetic CJD | E200K | MM | - | Canada | Brain & CSF |
| 66 | genetic CJD | E200K | MM | - | Canada | Brain & CSF |
| 67 | genetic CJD | E200K | MV | - | Canada | Brain & CSF |
| 68 | genetic CJD | E200K | MV | - | Canada | Brain & CSF |
| 69 | Fatal Familial Insomnia | D178N | MV | - | Canada | Brain & CSF |
| 70 | Fatal Familial Insomnia | D178N | MV | - | Canada | Brain & CSF |
| 71 | Fatal Familial Insomnia | D178N | MM | - | Canada | CSF |
| 72 | Fatal Familial Insomnia | D178N | MM | - | Canada | CSF |
| 73 | genetic CJD | D178N | VV | - | Canada | Brain & CSF |
| 74 | genetic CJD | V210I | MM | - | Canada | Brain |
| 75 | genetic CJD | V210I | MM | - | Canada | Brain & CSF |
| 76 | Gerstmann-Stäussler Scheinker | P102L | MM | - | Canada | Brain |
| 77 | Gerstmann-Stäussler Scheinker | P102L | MM | - | Canada | Brain |
| 78 | Gerstmann-Stäussler Scheinker | P105S | MM | - | Canada | Brain & CSF |
| 79 | genetic CJD | 2-OPRI | MV | - | Canada | Brain & CSF |
| 80 | genetic CJD | 4-OPRI | MM | - | Canada | CSF |
| 81 | genetic CJD | 5-OPRI | VV | - | Canada | Brain & CSF |
| 82 | genetic CJD | 5-OPRI | VV | - | Canada | Brain & CSF |
| 83 | Definitive CJD | - | VV | Type 1 | Canada | Brain |
| 84 | Definitive CJD | - | VV | Type 1 | Canada | Brain |
| 85 | Variably Protease-Sensitive Prionopathy | - | VV | - | Canada | Brain |
| 86 | Variably Protease-Sensitive Prionopathy | - | VV | - | Canada | Brain |
| 87 | Variably Protease-Sensitive Prionopathy | - | MM | - | Canada | Brain |
| 88 | Gerstmann-Stäussler Scheinker | P102L |  | - | USA | CSF |
| 89 | Gerstmann-Stäussler Scheinker | P102L |  | - | USA | CSF |
| 90 | Gerstmann-Stäussler Scheinker | P102L |  | - | USA | CSF |
| 91 | Gerstmann-Stäussler Scheinker | P102L |  | - | USA | CSF |
| 92 | Gerstmann-Stäussler Scheinker | P102L |  | - | USA | CSF |
| 93 | Gerstmann-Stäussler Scheinker | P102L |  | - | USA | CSF |
| 94 | Gerstmann-Stäussler Scheinker | P102L |  | - | USA | CSF |
| 94 | Gerstmann-Stäussler Scheinker | P102L |  | - | USA | CSF |
| 95 | Gerstmann-Stäussler Scheinker | P102L |  | - | USA | CSF |
| 97 | Gerstmann-Stäussler Scheinker | P102L |  | - | USA | CSF |
| 98 | Gerstmann-Stäussler Scheinker | A117V |  | - | USA | CSF |
| 99 | Fatal Familial Insomnia | D178N | MM | - | USA | CSF |
| 100 | Fatal Familial Insomnia | D178N | MM | - | USA | CSF |
| 101 | Fatal Familial Insomnia | D178N | MM | - | USA | CSF |
| 102 | Fatal Familial Insomnia | D178N | MM | - | USA | CSF |
| 103 | Fatal Familial Insomnia | D178N | MM | - | USA | CSF |
| 104 | Fatal Familial Insomnia | D178N | MM | - | USA | CSF |
| 105 | Fatal Familial Insomnia | D178N | MM | - | USA | CSF |
| 106 | Fatal Familial Insomnia | D178N | MM | - | USA | CSF |
| 107 | Fatal Familial Insomnia | D178N | MM | - | USA | CSF |
| 108 | Fatal Familial Insomnia | D178N | MM | - | USA | CSF |
| 109 | Genetic CJD | V180I | VV | - | USA | CSF |
| 110 | Variably Protease-Sensitive Prionopathy | - | MM | - | USA | CSF |
| 111 | Variably Protease-Sensitive Prionopathy | - | VV | - | USA | CSF |
| 112 | Variably Protease-Sensitive Prionopathy | - | VV | - | USA | CSF |
| 113 | Variably Protease-Sensitive Prionopathy | - | VV | - | USA | CSF |
| 114 | Variably Protease-Sensitive Prionopathy | - | MV | - | USA | CSF |
| 115 | Definitive sCJD | - | MM | Type 2 | USA | CSF |
| 116 | Definitive sCJD | - | MM | Type 2 | USA | CSF |
| 117 | Definitive sCJD | - | MM | Type 2 | USA | CSF |
| 118 | Definitive sCJD | - | MM | Type 2 | USA | CSF |
| 119 | Definitive sCJD | - | MM | Type 2 | USA | CSF |
| 120 | Definitive sCJD | - | VV | Type 1 | USA | CSF |
| 121 | Definitive sCJD | - | VV | Type 1 | USA | CSF |
| 122 | Definitive sCJD | - | VV | Type 1 | USA | CSF |
| 123 | Definitive sCJD | - | VV | Type 1 | USA | CSF |
| 124 | Definitive sCJD | - | VV | Type 1 | USA | CSF |

Supplemental Table 2: Individual QuIC test results from CSF samples for inherited prion disease and rare sCJD subtype cases, using various substrates for EP-QuIC, IQ RT-QuIC, and RT-QuIC.

|  |  | **EP-QuIC** | | | | **IQ RT-QuIC** | **RT-QuIC** | |
| --- | --- | --- | --- | --- | --- | --- | --- | --- |
| **Prion Disease** | **Case Number** | Deer Mouse | | FL Hamster | | Tr Hamster | Deer Mouse | FL Hamster |
|  |  | *15ul* | *30ul* | *15ul* | *30ul* |  |  |  |
|  | 62 | 3/3 | - | 3/3 | - | 3/3 | - | - |
| **gCJD - E200K** | 63 | 3/3 | - | 3/3 | - | 3/3 | 3/3 | 3/3 |
|  | 64 | 3/3 | - | 3/3 | - | 3/3 | 3/3 | 3/3 |
|  | 65 | 3/3 | - | 3/3 | - | 3/3 | 3/3 | 3/3 |
|  | 66 | 3/3 | - | 3/3 | - | 3/3 | 3/3 | 3/3 |
|  | 67 | 3/3 | - | 3/3 | - | 3/3 | 3/3 | 3/3 |
|  | 68 | 3/3 | - | 3/3 | - | 3/3 | 3/3 | 3/3 |
| **FFI - D178N** | 69 | 3/3 | - | 3/3 | - | 0/3 | 3/3 | 2/3 |
|  | 70 | 3/3 | - | 0/3 | - | 0/3 | 0/3 | 0/3 |
|  | 71 | 3/3 | - | 0/3 | - | 0/3 | 0/3 | 0/3 |
| **gCJD - D178N** | 73 | 3/3 | - | 2/3 | 2/3 | 0/3 | 2/3 | 1/3 |
| **gCJD V210I** | 74 | 3/3 | - | 3/3 | - | 3/3 | 3/3 | 3/3 |
|  | 75 | 3/3 | - | 3/3 | - | 2/3 | 3/3 | 3/3 |
| **GSS - P105S** | 78 | 3/3 | - | 3/3 | - | 0/3 | 3/3 | 3/3 |
| **2- OPRI** | 79 | 3/3 | - | 3/3 | - | 3/3 | 3/3 | 3/3 |
| **4- OPRI** | 80 | 3/3 | - | 3/3 | - | 3/3 | 3/3 | 3/3 |
| **5- OPRI** | 81 | 3/3 | - | 3/3 | - | 3/3 | 2/3 | 3/3 |
| **6- OPRI** | 82 | 3/3 | - | 3/3 | - | 3/3 | 3/3 | 3/3 |
| **GSS -P102L** | 88 | 3/3 | - | 3/3 | - | 0/3 | - | - |
|  | 89 | 2/3 | 3/3 | 3/3 | - | 0/3 | - | - |
|  | 90 | 1/3 | 2/3 | 0/3 | - | 0/3 | - | - |
|  | 91 | 3/3 | - | 3/3 | - | 3/3 | - | - |
|  | 92 | 3/3 | - | 3/3 | - | 0/3 | - | - |
|  | 93 | 1/3 | 2/3 | 0/3 | 0/3 | 0/3 | - | - |
|  | 94 | 3/3 | - | 3/3 | - | 3/3 | - | - |
|  | 95 | 3/3 | - | 3/3 | - | 3/3 | - | - |
|  | 96 | 3/3 | - | 3/3 | - | 0/3 | - | - |
|  | 97 | 3/3 | - | 3/3 | - | 3/3 | - | - |
| **GSS - A117V** | 98 | 3/3 | 3/3 | 0/3 | - | 0/3 | - | - |
| **FFI - D178N** | 99 | 1/3 | 3/3 | 0/3 | - | 0/3 | - | - |
|  | 100 | 2/3 | 3/3 | 1/3 | 1/3 | 0/3 | - | - |
|  | 101 | 3/3 | - | 3/3 | - | 0/3 | - | - |
|  | 102 | 3/3 | - | 3/3 | - | 0/3 | - | - |
|  | 103 | 1/3 | 3/3 | 0/3 | - | 0/3 | - | - |
|  | 104 | 2/3 | 2/3 | 0/3 | - | 0/3 | - | - |
|  | 105 | 3/3 | 3/3 | 2/3 | 2/3 | 0/3 | - | - |
|  | 106 | 2/3 | 3/3 | 0/3 | - | 0/3 | - | - |
|  | 107 | 3/3 | 3/3 | 0/3 | - | 0/3 | - | - |
|  | 108 | 1/3 | 1/3 | 0/3 | - | 0/3 | - | - |
| **gCJD - V180I** | 109 | 0/3 | - | 0/3 | - | 2/3 | - | - |
| **VPSPr** | 110 | 1/3 | 1/3 | 0/3 | 0/3 | 3/3 | - | - |
|  | 111 | 3/3 | - | 3/3 | - | 3/3 | - | - |
|  | 112 | 3/3 | - | 3/3 | - | 1/3 | - | - |
|  | 113 | 3/3 | - | 0/3 | - | 0/3 | - | - |
|  | 114 | 0/3 | - | 2/3 | 3/3 | 0/3* | - | - |
| **sCJD MM2** | 115 | 3/3 | - | 3/3 | - | 1/3 | - | - |
|  | 116 | 3/3 | - | 3/3 | - | 2/3 | - | - |
|  | 117 | 3/3 | - | 3/3 | - | 2/3 | - | - |
|  | 118 | 3/3 | - | 3/3 | - | 2/3 | - | - |
|  | 119 | 3/3 | - | 3/3 | - | 1/3 | - | - |
| **sCJD VV1** | 120 | 3/3 | - | 3/3 | - | 2/3 | - | - |
|  | 121 | 3/3 | - | 3/3 | - | 0/3 | - | - |
|  | 122 | 3/3 | - | 3/3 | - | 0/3 | - | - |
|  | 123 | 3/3 | - | 3/3 | - | 1/3 | - | - |
|  | 124 | 3/3 | - | 3/3 | - | 3/3 | - | - |

Supplemental Table 3: Statistical Analysis of CSF Substrate Performance in Genetic and Atypical Prion Diseases.

| **Diagnosis Group** | **DM vs. FL Ham** | **DM vs. Tr Ham** | **FL Ham vs. Tr Ham** | **Summary** |
| --- | --- | --- | --- | --- |
| FFI | **0.0036** | **< 0.0001** | 0.0957 | DM > FL > Tr |
| GSS | 0.2174 | **0.0014** | 0.0995 | DM > FL > Tr |
| VV1 | 1.0 | 0.1667 | 0.1667 | DM = FL > Tr |
| MM2 | 1.0 | 0.4444 | 0.4444 | DM = FL > Tr |
| VPSPr | 1.0 | 1.0 | 1.0 | No differences |
| gCJD | 1.0 | 1.0 | 1.0 | No differences |

Note: Significant p-values (P < 0.05) shown in bold. Two-tailed Fisher’s exact test.
